# Where the plasmids roam: large-scale sequence analysis reveals plasmids with large host ranges

**DOI:** 10.1099/mgen.0.000244

**Published:** 2019-01-09

**Authors:** Lauren Elisabeth Brooks, Mo Kaze, Mark Sistrom

**Affiliations:** ^1^​ University of California, Merced. 5200 N. Lake Road, Merced, CA 95343, USA; ^2^​ Utah Valley University, 800 W. University Parkway, Orem, UT 84058, USA

**Keywords:** plasmids, horizontal gene transfer, host range

## Abstract

Describing the role of plasmids and their contribution to the exchange of genetic material among bacteria is essential for understanding the fields of plasmid epidemiology, microbial ecology, and commercial and synthetic microbiology. Broad-host-range (BHR) plasmids are those that are found not only in a single bacterial species, but in members of different taxonomic groups and are of significant interest to researchers in many fields. We applied a novel approach to computationally identify new BHR plasmids, in which we searched for highly similar cognate plasmids within a comprehensive plasmid database. After identifying 125 plasmid groups with highly similar cognates found in multiple taxa, we closely examined BHR plasmids found in multiple families. The majority of our identified BHR plasmids are found in members of the *Enterobacteriaceae* and closely related taxa, while three BHR plasmids of potential commercial significance were found in two species of *Cyanobacteria*. One plasmid with an exceptionally broad host range was found in both Gram-positive and Gram-negative bacterial species. This analysis demonstrates the utility of this method in identifying new BHR plasmids while highlighting unknown ranges of previously documented plasmids.

## Data Summary

Data have been uploaded to Figshare (https://figshare.com/s/f341142916578e968fd3) including Tables S1 and S2 (available in the online version of this article).

Table S1: Metadata table for the comprehensive database

Table S2: Filtered blast output from the comprehensive database (SL_PlasmidDBOutput.txt – Headings are direct from blast and are ordered as follows: qseqid, sseqid, qlen, length, slen, pident, nident, mismatch, evalue, qcov and gaps)

**Impact Statement**

Identification of new broad-host-range (BHR) plasmids and their host ranges is important for a range of clinical, environmental and biotechnical applications. Identification of new BHR plasmids provides new targets for the development of tools for commercial and clinical applications, and increases our understanding of plasmid epidemiology and ecology in a range of environments. By taking a sequence-based approach, looking for similar plasmid sequences found in isolates from different taxonomic groupings, we take a top-down approach to look for possible BHR plasmids, rather than exploring which plasmids are transmissible given a specific host. This approach allows identification of plasmids that had not previously been reported as having a broad host range, thus providing a valuable resource from which to build upon with further examination and testing.

## Introduction

Genetic exchange outside of the traditional parent-to-offspring motif is an integral component of environmental adaptation and evolution in bacteria [1], and is often accomplished through the exchange of plasmids [2]. While selfish plasmids have been documented [3], this exchange of genetic material is often associated with benefits to the host as it can lead to the spread of a variety of functions, including degradation of hydrocarbons and anthropogenic toxic waste [4], bacteriocin and toxin production to ward off predators [5], and, alarmingly, antibiotic resistance and virulence plasmids that nullify the effectiveness of antibiotics and lead to novel and untreatable diseases [6]. Broad-host-range (BHR) plasmids are able to not only move between individuals within the same species, but also to replicate and move across more distantly related taxa, potentially transferring new functions to other species, families or classes of bacteria. Due to this wide range of possible hosts, BHR plasmids are of both clinical and environmental importance [7, 8] and commercial interest, as the identification of the replication machinery of BHR plasmids allows for the development of novel cloning vectors and synthetic plasmids [9].

Despite extensive academic and industrial interest in BHR plasmids, there are few definitions for what establishes a plasmid as being BHR. Since early attempts to explain the host ranges of specific plasmids [7], there have been attempts to define what determines whether a host range, or the variety of micro-organisms a plasmid can be maintained within and actively replicated within [10], can be classified as ‘broad’, ranging from specifically being able to be exchanged between *Enterobacteria* and *Pseudomonas* [11], to more broadly being able to cross taxonomic barriers [12]. Characteristics of BHR plasmids have been described, and recently reviewed by Jain and Srivastava [10], but these defining features are based on previously determined BHR plasmids, potentially neglecting features of currently unidentified groupings.

As the bulk of plasmid research and database compilation has centred on clinically relevant taxa, primarily from *Enterobacteriaceae* [13, 14], this probably generates bias in the characterization of BHR plasmids towards those present in these groups. For example, efforts have been undertaken to identify nucleotide sequences for several plasmids [15–17], which could define a plasmid as BHR and enable the use of sequence-based methods to search for the presence of BHR plasmids in a variety of environments [18, 19]. However, given the taxonomic biases in the currently identified BHR plasmids, these approaches may also be biased towards BHR plasmids found in those taxa and exclude additional sequences that could be relevant in many environments.

Given the ever-increasing number of publicly available sequences, and the recent update to the NCBI Plasmid Genome database, we opted to take a sequence-similarity-based approach to search for previously unknown, putative BHR plasmids. We compiled and examined a comprehensive plasmid database consisting of plasmids isolated from all taxonomic groupings. We focused on individual ‘cognate’ plasmids, defined here as plasmids with a highly similar sequence composition and found in multiple bacterial isolates. We identified and described the genetic content of cognate plasmids found in different taxa that are potentially new BHR plasmids. Using this method, we identified plasmids with host ranges spanning taxonomic levels including classes, families, genera and species. In our discussion, we have focused only on those plasmids found in multiple families while providing the data for the other identified BHR plasmids (Tables S1 and S2).

## Methods

### NCBI plasmid genomes

To compile a comprehensive plasmid database, we started with the recent NCBI Genomes update, which has a separate collection of plasmids as organisms. Compressed FASTA files containing plasmid ‘genome’ sequences were downloaded on 3 May 2018 from https://ftp.ncbi.nlm.nih.gov/refseq/release/plasmid resulting in 11 677 separate sequences. Using the R package Rentrez [20], we pulled the metadata available from the Nucleotide database for each entry based on the Locus ID contained in the header file for each plasmid. When available, metadata from the Bioproject, Biosample and Assembly databases were also pulled for each plasmid sequence.

We reviewed the metadata available for the downloaded sequences to screen for complete, assembled and correctly identified plasmid sequences. Of the 11 677 sequences downloaded, only 9763 were labelled as complete plasmids (i.e. the title did not indicate it was only a gene or a partial plasmid), 7434 of which indicated that the assembly was completed. Additionally, eight sequences described as phages were found and removed from inclusion in the database, resulting in 7426 plasmids following this initial screening.

### NCBI complete bacterial genomes

In addition to the pre-defined plasmid database, we compiled plasmids for incorporation into the database by extracting plasmid sequences from bacterial genomes with complete assemblies in NCBI’s Prokaryotic Genome database (https://www.ncbi.nlm.nih.gov/genome/browse#!/prokaryotes/) with a filter set for only bacterial genomes. Genomic assemblies labelled as partially complete or in contigs were not included, ensuring only completely assembled plasmid sequences were included. Sequences that were already included as part of the original Plasmid Genomes download, as identified by their accession or locus ID numbers, were removed as duplicates. This allowed us to include an additional 3466 plasmid sequences for analyses, resulting in 10 892 complete plasmid sequences.

### Metadata compilation for the comprehensive database

The two datasets described above were combined to give the comprehensive plasmids database used for all subsequent analyses (File S1). Metadata for this final list were compiled using the accession version number provided in the header for each plasmid sequence as described above (Table S1).

### Rep and MOB typing

Although options exist for plasmid typing, the replicon (Rep) [13, 19] and MOB (i.e. mobility as determined by the encoded relaxase) [21, 22] typing schemes have been developed and tested on a limited taxonomic range. For Rep typing, plasmid sequences were queried against a local copy of the replicon typing database for both the *Enterobacteriaceae* and Gram-positive PlasmidFinder databases [23] and screened to only include hits with a percentage identity of 80 % and a coverage cutoff of 60 %.

Mobility typing was accomplished following the method developed by Orlek *et al.* [14]. The GitHub repository containing all necessary scripts was cloned for local storage and the parent script was used to type each plasmid in the comprehensive database.

### Identification of cognate plasmids

To assess whether a plasmid was found in multiple entries, termed here as a cognate plasmid, we conducted a local nucleotide blast search for each plasmid in the database against a concatenated version of the comprehensive database, allowing only one high scoring pair (HSP) per sequence. The complete list of hits identified by blast was examined to identify natural breaks in the data that would provide natural stopping points (Fig. S1). However, no clear breaks could be identified, so we elected to restrict our potential cognates to only those that had greater than 80 % sequence coverage, and the subject and query length were similar (within 80 %). This resulted in a minimum sequence similarity of 75 % identical.

### Network analysis to determine the host range for each group of cognates

Once a list of blast hits meeting the requirements determined for consideration as cognates was compiled (Table S2), the identified plasmid sequences were used to compute a network to demonstrate the connections between similar plasmid sequences using the Network package in R [24]. This network consisted of nodes (or bacterial isolates) connected to one another based upon whether sufficient sequence similarity was detected. Metadata from the comprehensive database for each subject and query were used to identify the potential range of the plasmids. We used this determined range of each plasmid to compile a list of BHR plasmids, which are defined for this paper as those plasmids found in multiple taxonomic groups. Visualization of the network was conducted with tools from the additional packages igraph [25], tidygraph [26], ggraph [27], visNetwork [28] and networkD3 [29].

### Coding sequence identification and classification

To explore gene families that are dominant among the BHR plasmids, we annotated the identified plasmids using Prokka [30]. Although coding sequences identified by the original publisher of the sequences are available, we chose to re-annotate the genes using a single software package for consistency. Identified coding sequences from Prokka were then input into the eggNOG Mapper [31] to construct gene ontology predictions for each plasmid, allowing us to explore the primary functions associated with each plasmid.

### blast of identified BHR plasmids against the nucleotide collection

Construction of the comprehensive plasmid database was conducted in a conservative way to ensure only complete plasmid sequences were included. It is incapable of detecting matches for these sequences outside of this database. To check for matches to our determined BHR plasmids in all published sequences, we used blast to compare the identified BHR sequences against the Nucleotide database on NCBI. Default settings on blast were not altered, and results were screened for hits having at least 80 % of the query coverage to identify additional cognates.

## Results and Discussion

### Exploring the ranges and composition of cognate plasmids

Identification of new BHR plasmids and their host ranges provides insight into adaptation and evolution and expands our tools for bioengineering novel cloning vectors, and microbial-based innovations for bioproducts, biofuels and bioremediation. Taking a sequence-based approach to searching for new BHR plasmids, we compiled and examined a comprehensive database of bacterial plasmids to examine their host range. Cognate plasmids were identified based on high sequence similarity shared between a group of plasmids. Of the 10 892 plasmids, approximately half (5549) had no identified cognates, while 5343 were found to have cognates within the database. Of those found in more than one sequence entry, these were grouped into 2017 distinct nodes, 125 of which were found in entries from different species and 52 of which spanned members of different genera (File S1).

**Fig. 1.**
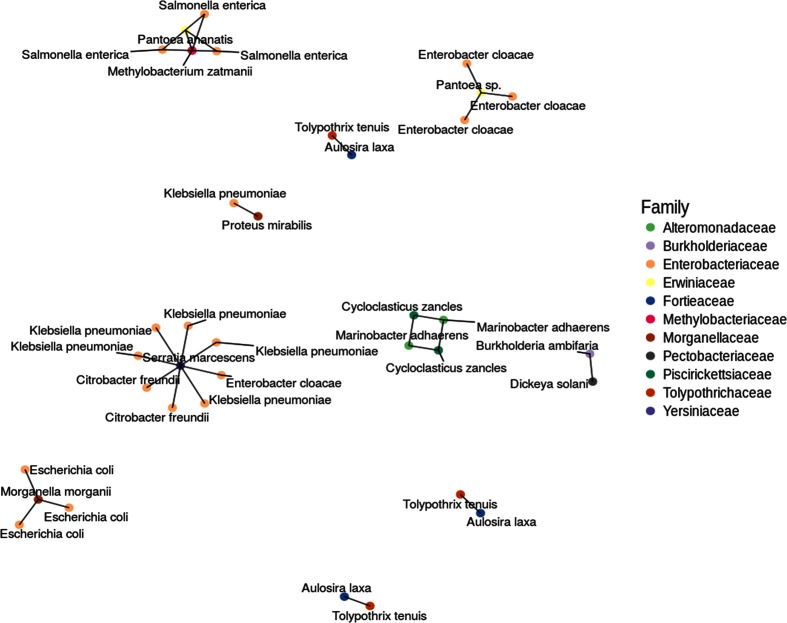
Network diagram showing the ten inter-family cognate plasmids considered of broad host range.

Here we highlight the composition and characteristics of BHR plasmids, focusing only on those plasmids for which cognate plasmids were found in different families (Fig. 1), resulting in ten unique plasmids with a range of features and characteristics (Table 1). While plasmids were initially compared to each other only within the compiled comprehensive database to identify potential BHR plasmids, we acknowledge that this conservative approach will fail to identify all highly similar sequences. Thus, once we compiled a list of putative BHR plasmids, a representative sequence was selected and blast was used to identify additional nucleotide sequences from the entire NCBI nucleotide collection with high similarity, the results of which are included in the discussion below.

**Table 1.** Characteristics of identified BHR plasmids identified using the comprehensive plasmid database

Plasmid group	Plasmid name (previously reported)	NCBI accession IDs	Replicon type	MOB type	Plasmid length	Coding sequences	Phylum	Species (family)	Original references
pBII_1	pBII_1 Plasmid unnamed	CP009797.1 CP017455.1	IncP	MOBP	43 kb	40 or 41	Proteobacteria	*Burkholderia ambifaria* (*Burkholderiaceae*) *Dickeya solani* (*Pectobacteriaceae*)	[32] [33]
pMACZ	pHP-42 p7ME01	CP001979.1 CP006601.1		MOBP	42 kb	37	Proteobacteria	*Marinobacter adhaerens* HP15 (*Alteromonadaceae*) *Cycloclasticus zancles* 78-ME (*Piscirickettsiaceae*)	[38] [39]
pKPC-47e	pKPC-47e pKPC-47e pKPC-47e pKPC-1c5	CP008901.1 CP008908.1 CP009858.1 CP009881.1	IncN	MOBF	50 kb	35 or 36	Proteobacteria	*Enterobacter cloacae* ECNIH3 (*Enterobacteriaceae*) *Enterobacter cloacae* ECR091 (*Enterobacteriaceae*) *Enterobacter cloacae* (*Enterobacteriaceae*) *Pantoea* sp. PSNIH1 (*Erwiniaceae*)	[41] [41] [41] [41]
pMS6671c	Unnamed-1 C_Kpneumoniae_MS6671	CP026060.1 LN824136.1		MOBF	34.6 kb	26	*Proteobacteria*	*Proteus mirabilis* (*Morganellaceae*) *Klebsiella pneumoniae* (*Enterobacteriaceae*)	[44] [43]
pTTAL_1	plasmid2 plasmid3	NZ_AP018250.1 NZ_AP018310.1		MOBP	216 kb	119	*Cyanobacteria*	*Tolypothrix tenuis* PCC 7101 (*Tolypothrichaceae*) *Aulosira laxa* NIES-50 (*Fortieaceae*)	BioProject ID: PRJDB5665 BioProject ID: PRJDB5665
pTTAL_2	plasmid3 plasmid4	NZ_AP018251.1 NZ_AP018311.1			56 kb	28 or 29	*Cyanobacteria*	*Tolypothrix tenuis* PCC 7101 (Tolypothrichaceae) *Aulosira laxa* NIES-50 (*Fortieaceae*)	BioProject ID: PRJDB5665 BioProject ID: PRJDB5665
pTTAL_3	plasmid4 plasmid5	NZ_AP018252.1 NZ_AP018312.1		MOBF	46 kb	24 or 25	*Cyanobacteria*	*Tolypothrix tenuis* PCC 7101 (*Tolypothrichaceae*) *Aulosira laxa* NIES-50 (*Fortieaceae*)	BioProject ID: PRJDB5665 BioProject ID: PRJDB5665
pCAV	pKP13b pCAV1311-3223 pCAV1321-3233 pCAV1492-3233 pCAV1741-3223 pCAV1042-3223 unnamed5 unnamed5 unnamed8	CP003994.1 CP011569.1 CP011604.1 CP011637.1 CP011652.1 CP018666.1 CP024487.1 CP024494.1 NZ_CP024514.1		MOBP	3.2 kb	3 or 4	*Proteobacteria*	*Klebsiella pneumoniae* subsp*. pneumoniae* Kp13 (*Enterobacteriaceae*) *Enterobacter cloacae* (*Enterobacteriaceae*) *Citrobacter freundii* (*Enterobacteriaceae*) *Serratia marcescens* (*Yersiniaceae*) *Citrobacter freundii* (*Enterobacteriaceae*) *Klebsiella pneumoniae* (*Enterobacteriaceae*) *Klebsiella pneumoniae* (*Enterobacteriaceae*) *Klebsiella pneumoniae* (*Enterobacteriaceae*) *Klebsiella pneumoniae* (*Enterobacteriaceae*)	[53] [53] [53] [53] [54] [54] [52] [52] [52]
pECMM	pMNCRE44_1 pECAZ155_4 pEC881_8 unnamed	CP010877.1 CP019004.1 CP019021.1 CP023506.1	Col(MG828)			1.5 kb	*Proteobacteria*	*Escherichia coli* (*Enterobacteriaceae*) *Escherichia coli* (*Enterobacteriaceae*) *Escherichia coli* (*Enterobacteriaceae*) *Morganella morganii* (*Morganellaceae*)	[55] [56] [56] [44]
pMPSE	Unnamed P_unamed4 Unnamed2 Unnamed2 Unnamed1	CP021055.1 CP022430.1 CP022493.1 CP022496.1 CP022501.1				1.8 kb	*Proteobacteia*	*Methylobacterium zatmanii* (*Methylobacteriaceae*) *Pantoea ananatis* (*Erwiniaceae*) *Salmonella enterica* subsp*. enterica* serovar *Saintpaul* (*Enterobacteriaceae*) *Salmonella enterica* subsp*. enterica* serovar *Derby* (*Enterobacteriaceae*) *Salmonella enterica* subsp*. enterica* serovar *Kentucky* str. SA20030505 (*Enterobacteriaceae*)	BioProject ID: PRJNA376590 [59] [57] [57] [57]

### One extremely BHR plasmid was found to have cognates in entries spanning multiple phyla

**Fig. 3.**
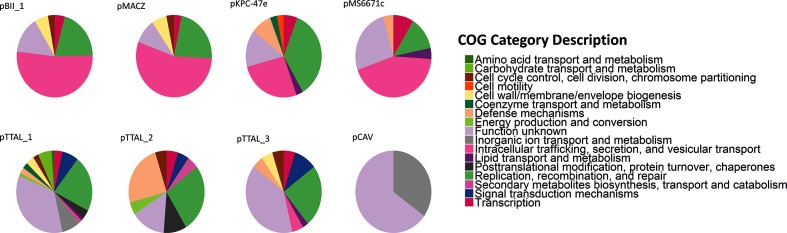
Expanded cladogram including the nucleotide collection blast good hits for pBCII_1 shows the wide range of organisms found to contain highly similar cognates of this plasmid.

In our initial analysis using only plasmids in the compiled database, plasmid pBCII_1 [32] was found in members of both *Betaproteobacteria*, *Burkholderia ambifaria* AMMD [32], and *Gammaproteobacteria*, *Dickeya solani* ([33]; Table 1). The host range of this plasmid was expanded to include two members from the phylum *Actinobacteria* as well as additional representatives from the classes *Alphaproteobacteria*, *Betaproteobacteria* and *Gammaproteobacteria* of the *Proteobacteria* (Fig. 3) when incorporating additional sequences from the nucleotide collection sequences. While most plasmids replicate within a specific genus or family [34], the pBCII_1 plasmid group contains members from different phyla including both the Gram-positive *Actinobacteria* and Gram-negative *Proteobacteria*, which although previously observed, remains rare [35].

**Fig. 2.**
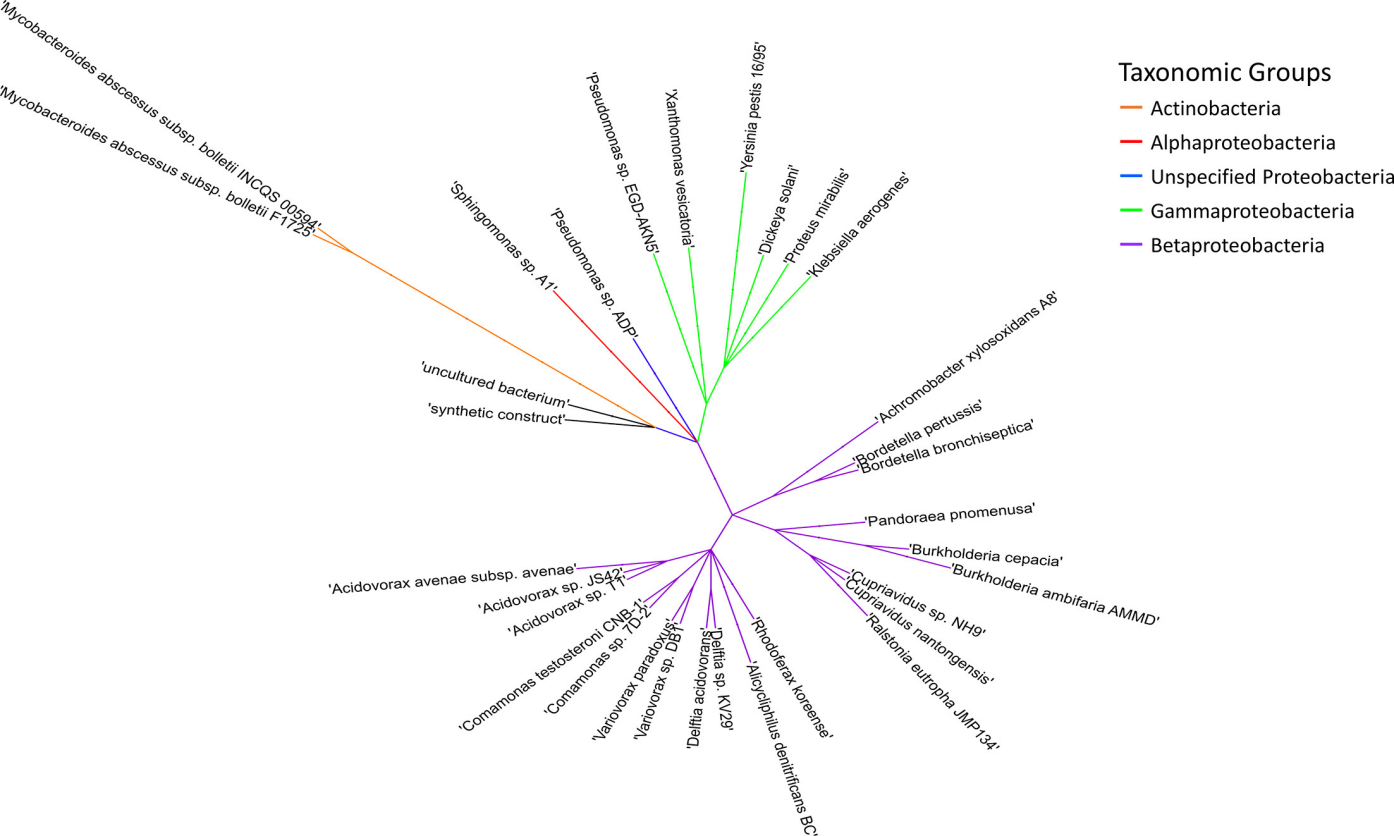
Pie charts showing relative counts of gene content for all BHR plasmids showing a range of functions. Although there was a large amount of variation across the plasmids, the dominant COG groups were intracellular trafficking, secretion and vesicular transport; replication, recombination and repair; and unknown function.

All occurrences of this 43 kb plasmid were successfully Rep-and MOB-typed as IncP and MOBP, respectively. COG functions for the annotated 40 coding sequences identified on the plasmid were predominantly assigned to the categories of intracellular trafficking, secretion and vesicular transport (52 %), and replication, recombination and repair (20 %) with the remainder of the coding sequences assigned to an additional four COG categories (Fig. 2a).

Although more commonly thought of as being associated with Gram-negative bactieria [36], the occurrence of an IncP plasmid in Gram-positive *Mycobacterium abscessus* has been reported previously [37]. Our methodology detected the presence of the IncP pBCII_1 plasmid in the same isolate as previously reported, as well as a second member of *Mycobacterium abscessus* and other taxa potentially carrying and replicating this plasmid (Fig. 3; File S2). While this plasmid had previously been detected and the ability to replicate in new taxa established, our findings suggest that the plasmid may be capable of occurring in an even wider taxonomic group than previously believed. While this sequence-based search suggests that these are real plasmid sequences, further investigations to determine the ability to transfer among these hosts is necessary.

### A BHR plasmid found in separate orders was identified in sequences from oceanic bacteria *Marinobacter adhaerens* and *Cycloclasticus zancles*

This plasmid, found in isolates from two distinct orders of *Gammaproteobacteria*, with *Marinobacter adhaerens* [38] from the order *Alteromonadales* and *Cycloclasticus zancles* 78-ME [39] from the order *Triotrichales*, was approximately 42 kb in length and was found to have 37 putative coding sequences. The sequences from the distinct entries were found to be highly similar, with 92 % sequence identity across the 90 % (35 kb) of the plasmid genomes that were a match. While replicon typing with both the *Enterobacteriaceae* database and the Gram-positive database failed, the plasmid was successfully MOB-typed as having a relaxase from the family MOBP. Analysis of the coding sequences through EggNOG identified on this plasmid found that over half (54 %) were involved in intracellular trafficking, secretion and vesicular transport largely predicted to be conjugal transfer proteins, while replication, recombination and repair made up an additional 22 % of the identified coding sequences. Additional coding regions making up smaller portions of the genomes were also identified (Fig. 2b).

While additional blast searching of the nucleotide collection failed to provide additional information, the presence of this plasmid in two distantly related taxa from different locations (Table S1) suggests the possibility that this and potentially additional BHR plasmids could be present in oceans and other under-investigated environments, potentially contributing to key functions in these microbial communities. The essential role of plasmid exchange for some marine taxa has been documented [40] and the detection of this cognate plasmid in oceanic bacteria suggests this may be more common than currently known.

### Two antibiotic resistance encoding plasmids were found in members of the family *Enterobacteriaceae* and closely related families

A beta-lactamase encoding plasmid was found in three entries of *Enterobacter cloacae* and one of *Pantoea* species [41] and when including additional blast results has an expanded range including a cognate found in the human pathogen *Pseudomonas aeruginosa* (File S2). The 50 kb plasmid, previously named pKPC-47e [41], was successfully Rep- and MOB-typed as IncN and MOBF, respectively (Table 1). Additionally, this plasmid was found to encode resistance to penicillins, cephalosporins, cabapenems and monobactams through an identified beta-lactamase, KPC-1 [42]. The majority of the 35 coding sequences identified on each plasmid were classified as being involved in replication, recombination and repair (35 %) while 26 % were involved in intracellular trafficking (Fig. 2c).

Another BHR plasmid found in a carbapenem-resistant *Klebsiella pneumoniae* [43] was also found in *Proteus mirabilis* [44]. This 34 kb plasmid, originally described as the known beta-lactamase encoding plasmid MS6771 in *K. pneumoniae*, was identified as MOB type F with no identified replicon type. The 26 identified coding sequences were identified mainly from the intracellular trafficking, secretion and vesicular transport (43 %) and function unknown categories (Fig. 2d). The pCAV plasmid, found in a number of human pathogens as well as the soil bacterium *Citrobacter freundii*, was found in additional human pathogens as well as several common plant pathogens, all from the family *Enterobacteriaceae* or closely related taxa.

Both plasmids, isolated primarily from *Enterobaceriaceae*, and closely related or recently reclassified species, include clinically relevant antibiotic resistance genes and pose an increasing threat to human health [45]. While all entries containing these plasmids are considered common soil-dwellers, they are also clinically important pathogens including many normally occurring human gut bacteria that become opportunistic infections once outside their normal environments [46, 47]. Many have been isolated from nosocomial infections, indicating an environment in which plasmid exchange could occur. It is of great importance to expand upon the work done here to identify the host ranges of these plasmids and environments harbouring the bacteria carrying them, as there is the dangerous possibility of exchange of virulence and resistance plasmids.

### Three different cognate plasmids were found in cyanobacteria groups *Tolypothrix tenuis* and *Aulosira laxa*

Three distinct plasmids were found together in the same genome assemblies of cyanobacterial taxa *Tolypothrix tenuis* and *Aulosira laxa* (unpublished; BioProject ID: PRJDB5665). Two of the three plasmids were MOB-typed (MOBF and MOBP), while none had identifiable replicons (Table 1). The three groups of cognate plasmids had a wide range of lengths (46–216 kb) and contained between 24 and 119 coding sequences. While many of the functions for the plasmids were classified as unknown functional COG categories, replication, recombination and repair genes were frequent (Fig. 2e–g). In contrast to the other plasmids identified, these three cognate plasmids had a relatively wide range of COG categories.

BHR plasmids have been constructed using features of cyanobacterial plasmids and have been applied as various bioproducts: biofuels, plant hybrids, bioremediation, anti-cancer pharmaceuticals and agricultural soil additives [48–51]. However, the cyanobacterial plasmids identified here are little known, and failed to correspond to any known BHR plasmids used commercially, despite the wide host range identified here. With no identifiable replicon structure, further investigation into these plasmids as potential commercially significant vectors should be considered.

### Several plasmids with largely unknown functions were found in multiple families

No replicon was identified for the 3.2 kb plasmid, pCAV, despite most entries containing this plasmid being from *Enterobacteriaceae* [52–54], for which the replicon typing scheme was developed. MOB typing, however, identified the presence of a relaxase from the Type P family. Annotation of this relatively short plasmid showed that 65 % of the identified coding sequences had unknown function while 35 % were involved in inorganic ion transport and metabolism (Fig. 2h). The coding sequences annotated as transport were further identified as as a multidrug resistance protein involved in drug efflux.

Two additional groups of cognate plasmids had no identified coding sequences and no identifiable replicons or relaxases. The first plasmid, 1.5 kbp in length, was found in three entries of *Escherichia coli* [55, 56] and one *Morganella morganii* [44]. The second plasmid was found in three entries of *Salmonella enterica* [57, 58], one of *Pantoea annanatis* [59] and one of *Methylobacterium zatmanii* (unpublished; BioProject ID: PRJNA376590).

### Home on the range: conclusions from a new method of exploring host ranges

As with all studies that rely on the use of databases, our ability to detect BHR plasmids in the sequences is limited by the availability of data and the screening criteria we used to construct our comprehensive database. We sought to use conservative methods to ensure the integrity of our findings but acknowledge that this may exclude other findings of interest. Additionally, our methodology has the potential to result in a collection of cognate plasmids that may contain possible false positives that are highly similar only due to chance as well as false negatives that do in fact share common histories but were screened by our criteria. To enable the utility of this database and methodology to other researchers, we have made our compiled database available so that others may explore further and hope to provide a model for how this methodology could be used, not necessarily to set the standard for future researchers to follow.

Using our strict standards for inclusion, we still faced problems with incompleteness within the available data that limits our ability to identify BHR plasmids. The comprehensive plasmid database consisted of 10 892 complete plasmid sequences spanning 24 bacterial phyla, encompassing a total of 2169 unique NCBI taxonomic IDs. Metadata compiled for the sequences include all information from the nucleotide entry, taxonomic information compiled using the nucleotide taxonomic ID, and any successful plasmid replicon [13, 19] or mobility [21, 22] typing. However, incomplete metadata provided for plasmids in publicly available data prevented further analyses, such as examining the spread of plasmids across isolation sources. As noted previously, plasmids in public databases often have incomplete gene annotations and large proportions of pseudogenes [60], thus making it difficult to make statements about plasmid functions without first re-examining the sequences provided. This highlights the significance and essentiality that sequences entered into public databases include detailed metadata such as isolation source and location, phenotypic attributes if cultured, and habitat conditions.

Among the data that were available, there was a bias detected with respect to the included potential hosts. Within the broad range of taxa represented in the comprehensive database, there was a heavy skew towards plasmids from medically relevant groupings, particularly the family *Enterobacteriaceae*, thus potentially limiting our ability to identify potential BHR plasmids. Most sequences in the comprehensive database were from the phylum *Proteobacteria* (*n*=6355). A large abundance of these (*n*=2511) were found in members of the family *Enterobacteriaceae* and similar to the size of the exclusive *Enterobacteriaceae* database presented recently by Orlek *et al*. [14]. In addition to *Proteobacteria*, *Firmicutes* were also highly represented (*n*=2382), while no other phyla were represented by more than 1000 plasmid sequences. The second most represented phylum, *Firmicutes*, was surprisingly poorly represented in the BHR plasmids. This finding was unexpected as the Gram-positive classes *Bacilli* and *Clostridia* have well-characterized plasmids given their role in antibiotic resistance gene transmission via plasmids [61].

These newly identified BHR plasmids may have interesting new applications to commercial biotechnology and bioproducts, and the epidemiology of known and emerging pathogens, yet have been overlooked by traditional methods for searching for BHR plasmids. In light of the increasing amount of genomic data concerning plasmid composition and their host ranges, continued examination for newly identified cognate plasmids could continue to shape our understanding of host ranges and complement the traditional screening methods used for plasmid transmission. This method has already provided candidates to explore further as presented here, but also should continuously be employed as the number of published genome sequences continues to grow, providing novel potential replication systems and plasmid characteristics of interest that contribute to the expanded host ranges observed for BHR plasmids.

The findings of a significant number of plasmid groups being found in different species, genera and even families as highlighted here continue to call into question the consideration of plasmids as belonging to specific taxonomic groups. Recent papers highlighting the ability of plasmids [62, 63] to exist and replicate within wider than previously believed taxonomic ranges only opens the door to more explorations into what makes a BHR plasmid. Had we not considered all plasmids, but instead restricted our methods to databases encompassing only a taxonomic grouping of interest, we would have missed the opportunity to explore these unclassified BHR plasmids. Cognate plasmids found across multiple families, orders and classes must be taken into consideration along with their Rep and MOB typing when seeking to classify plasmids as they may not be specific to any taxonomic group, but instead are capable of being isolated from multiple taxa.

## Data bibliography

1. Brooks, Figshare, DOI: 10.1099/mgen.0.000244 (2018).

## Supplementary Data

Supplementary File 1

Supplementary File 2

Supplementary File 3
