# Supplementary material for: Where the plasmids roam: large-scale sequence analysis reveals plasmids with large host ranges
Source: Microb Genom. 2019 Jan 9;5(1):e000244. doi: 10.1099/mgen.0.000244 (PMC6412061; doi:10.1099/mgen.0.000244)
Supplement: Supplementary File 1 [file mgen-5-244-s001.pdf]

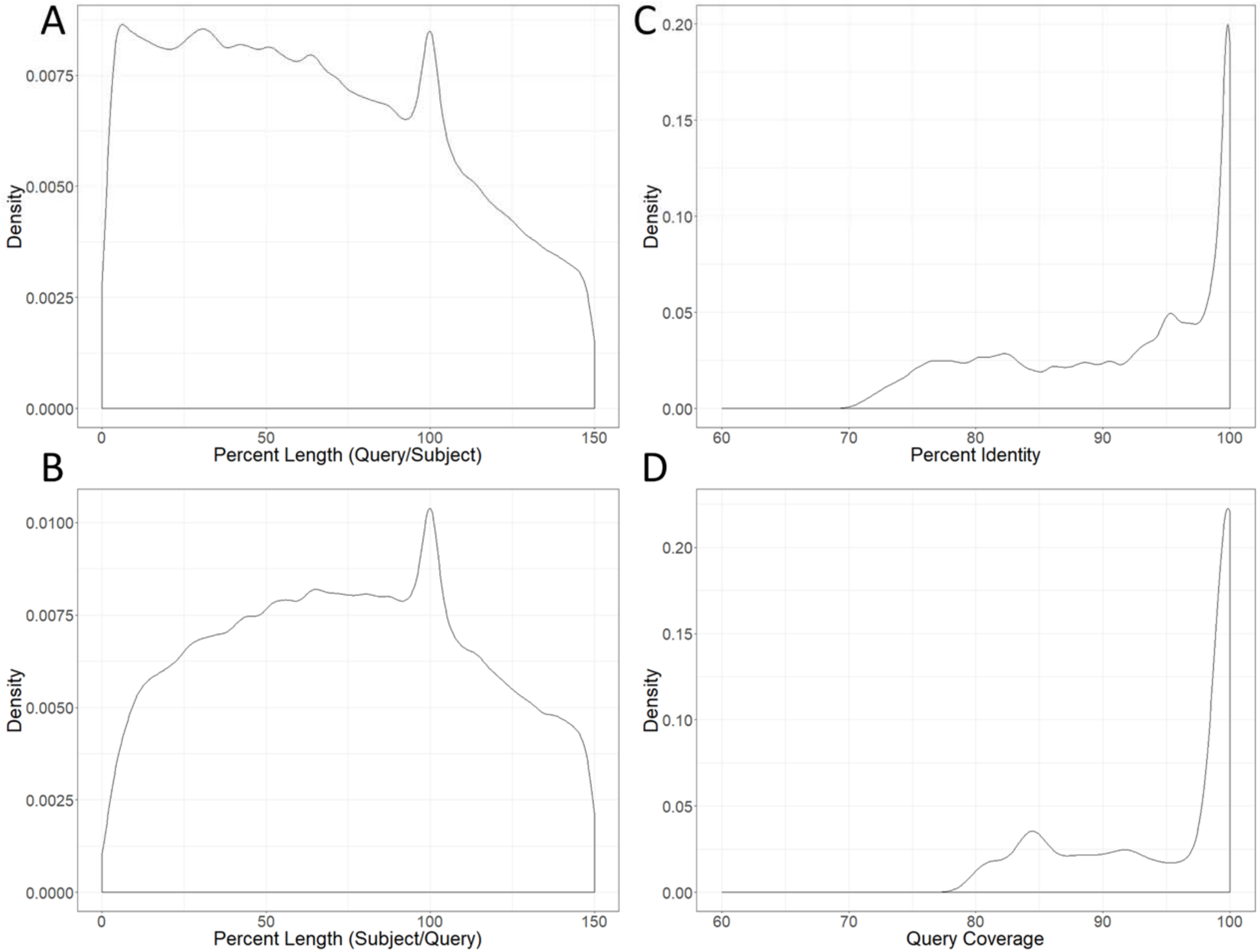

Supplemental Figure 1: Density plots for the screening criteria of BLAST output showing no clear break points to suggest cutoff values. A) The percent length of the raw BLAST output (Query length divided by Subject Length) B) The percent length of the raw BLAST output (Subject length divided by Query length). C) The Percent Identity values of the raw BLAST output. D) The Query coverage values of the final list of cognate plasmids, following filtering of the other criteria.
